# Reconciling conflicting clinical studies of antioxidant supplementation as HIV therapy: a mathematical approach

**DOI:** 10.1186/1471-2458-9-S1-S12

**Published:** 2009-11-18

**Authors:** Rolina D van Gaalen, Lindi M Wahl

**Affiliations:** 1 Department of Applied Mathematics, University of Western Ontario, London, Ontario, Canada

## Abstract

**Background:**

Small, highly reactive molecules called reactive oxygen species (ROS) play a crucial role in cell signalling and infection control. However, high levels of ROS can cause significant damage to cell structure and function. Studies have shown that infection with the human immunodeficiency virus (HIV) results in increased ROS concentrations, which can in turn lead to faster progression of HIV infection, and cause CD4^+ ^T-cell apoptosis. To counteract these effects, clinical studies have explored the possibility of raising antioxidant levels, with mixed results.

**Methods:**

In this paper, a mathematical model is used to explore this potential therapy, both analytically and numerically. For the numerical work, we use clinical data from both HIV-negative and HIV-positive injection drug users (IDUs) to estimate model parameters; these groups have lower baseline concentrations of antioxidants than non-IDU controls.

**Results:**

Our model suggests that increases in CD4^+ ^T cell concentrations can result from moderate levels of daily antioxidant supplementation, while excessive supplementation has the potential to cause periods of immunosuppression.

**Conclusion:**

We discuss implications for HIV therapy in IDUs and other populations which may have low baseline concentrations of antioxidants.

## Background

Reactive oxygen species (ROS) are highly reactive byproducts of cellular respiration. As second messengers, they play an important role in cell signaling and in gene regulation (e.g., cytokine, growth factor, and hormone action and secretion; ion transport; transcription; neuromodulation; and apoptosis) [1,2]. ROS are also important for the normal function of the immune system; T cells both are influenced by and influence intracellular ROS levels. In particular, ROS play a positive role in the proliferation of T cells and immunological defence [1-5].

A variety of reactive oxygen species are produced throughout the body. One particular species of interest, superoxide (), is generated in two ways and for different reasons [5]: (1) as an accidental result of incomplete electron transfers in the electron transport chain and (2) in activated white blood cells with the purpose of destroying pathogens. Moreover, upon production, these  molecules are rapidly metabolized into hydrogen peroxide (H_2_O_2_), a mild oxidant, which further helps to destroy some pathogens. Intermediate concentrations of H_2_O_2 _(and certain other ROS) result in the activation of nuclear factor *κ*B (NF-*κ*B), a transcription factor that upregulates several cellular processes, including cell proliferation and apoptosis [1,6,7].

Despite their positive role, reactive oxygen species can be harmful. At normal ROS concentrations, cell function and structure are protected from destructive interactions with ROS by various defence mechanisms. These include the use of both enzymatic and nonenzymatic antioxidants, substances that significantly delay or prevent the oxidation of a given substrate. Non-enzymatic antioxidants obtained directly from the diet (i.e., glutathione, vitamins A, C and E, and flavenoids) decrease oxygen concentrations, remove catalytic metal ions and eliminate radicals from the system [8,9]. Enzymatic antioxidants remove ROS from the system and are not consumed by the reaction. These enzymes, such as superoxide dismutases, catalase and glutathione peroxide, are naturally produced by the body [9]; oral supplements and injections are also available [9]. In addition, antioxidants repair oxidative damage, eliminate damaged molecules and prevent mutations from occurring [8].

In the event that intracellular ROS levels increase moderately, cells respond by boosting antioxidant levels and by promoting proinflammatory gene expression [10,11]. There are two main functions of the resulting translated proteins: (1) signaling proteins activate the immune system by various cytokines, growth factors and chemokines, and (2) enzymes improve a cell's response to inflammatory, growth-stimulatory and apoptotic signals [11]. When ROS levels exceed a cell's antioxidant capacity, oxidative stress is reached; this has the potential to cause significant damage to DNA, proteins and lipids, and can induce apoptosis. In addition, conditions favourable for the pathogenesis of several diseases may be created [1]. Such high levels of ROS are generally the result of chronic and acute inflammatory diseases or environmental stress [10].

Individuals infected by the human immunodeficiency virus (HIV) exhibit heightened serum concentrations of ROS [6,10,12,13] and lowered antioxidant concentrations [14]. The resulting oxidative stress affects disease progression in several ways. First, oxidative damage to CD4^+ ^T cells may impair the immune system's response to HIV [15]. Second, the well-known hallmark of HIV, the depletion of CD4^+ ^T cell concentration in the plasma, is further exacerbated by oxidative stress-induced apoptosis. Third, increased HIV transcription leading to faster disease progression results from an increased activation of NF-*κ*B [6]. It has been found that while NF-*κ*B activation is not absolutely necessary for viral replication, it accelerates the process 20-fold [15-17]. Moreover, it has been suggested that NF-*κ*B is itself activated by HIV [16]. It has been shown that this activation of NF-*κ*B is inhibited by antioxidants (such as N-acetyl cysteine and pyrrolidine dithiocarbarnate) [7].

The lowered antioxidant concentrations observed in HIV-positive individuals are associated with micronutrient deficiencies [14,18] which are themselves caused by a combination of decreased nutrient intake, gastrointestinal malabsorption, increased nutritional requirements, and psychosocial factors [18,19]. Observational studies and intervention trials of nutritional shortfalls in HIV-positive individuals not receiving HAART reveal that low serum concentrations of micronutrients such as thiamine, selenium, zinc, and vitamins A, B-3, B-6, B-12, C, D, and E have been independently linked to a weakened immune system and a higher risk of the following: vertical transmission [20], faster disease progression [21], low CD4^+ ^T cell counts, HIV-related diseases, and mortality [22]. Intervention trials have shown that such individuals can benefit from micronutrient supplementation [22-24]. Among their other benefits, certain micronutrients have antioxidant properties: carotenoids and vitamins A, C, and E [5]. Since elevated ROS levels have been linked to more rapid HIV progression [13,25], antioxidant supplementation has been suggested [6,26] and studied [22,27-29] as a potential complement to HIV therapy.

Despite many indications that antioxidant supplementation is beneficial in HIV-positive individuals [22,27,28], it has been suggested that antioxidant supplementation may not be universally recommended [22]. For example, although reduced mortality has been shown in HIV-positive children receiving vitamin A supplementation [30], the administration of vitamin A supplements to women has been implicated in increased vaginal viral shedding (no effect on risk was observed from vitamins B, C, and E) [31], a heightened risk of mother-to-child HIV transmission [32], and hastened progression of child mortality [30]. In addition, high doses of vitamin C supplementation have been shown to reduce the bioavailability of the protease inhibitor, indinavir [33]. These findings, among others, undoubtedly necessitate concern, and have led authors to question the benefits of universal vitamin A supplementation for women in HIV-endemic areas [30,31]. Despite these concerns, Fawzi *et al. *[31] maintain that prenatal supplementation of vitamins B, C, and E should be continued due to their many reported positive effects on maternal and fetal health.

In short, studies have shown a range of potential implications of antioxidant supplementation. Some have found reasons for concern, others have shown negligible effects, and still others have been positive about the potential of antioxidant supplementation as a therapy or supplemental therapy for HIV-infected individuals. Despite this range of opinions, the 2007 review by Drain *et al. *[22] maintains that supplementation in individuals not receiving HAART is clearly beneficial; however, there are not sufficient data to indicate whether the same can be said for individuals receiving HAART.

Injection drug users form a particular group of interest due to the endemic nature of HIV infection in this population. According to the WHO, the global population of injection drug users (IDUs) consists of approximately 15.9 million people, of which 3 million are HIV-positive. The spread of the virus is particularly rampant in populations where injecting equipment is re-used and shared. Of the new HIV infections, one in ten are caused by the use of injection drugs. In Eastern Europe and Central Asia, drug use can be attributed to 80% of all HIV infections [34]. Thus, for the potential eradication of HIV, it is critical that this population, among others, be targeted. Furthermore, oxidative stress has been implicated as a factor in faster disease progression in HIV-positive IDUs. Lower serum concentrations of vitamin A, compared with HIV-negative IDUs, have also been observed [35].

A particular clinical study conducted by Jaruga *et al. *[28] demonstrated a clear benefit for antioxidant therapy in IDUs when compared with the appropriate control group. In this study, samples were collected from a control group of 10 healthy volunteers, a group of 15 HIV-negative injection drug users (denoted HIV(-)) and a group of 30 asymptomatic HIV-positive injection drug users (denoted HIV(+)). The latter HIV-positive group was divided into two subgroups: one subgroup of 15 patients received a placebo (HIV(+)P), while the other received a daily supplement of 5000 units of vitamin A, 100 units of vitamin E and 50 mg of vitamin C (HIV(+)V). After six consecutive months of treatment, it was found that patients in groups HIV(-) and HIV(+)P had significantly lower blood plasma concentrations of vitamins A, C and E than the control group, while individuals in the HIV(+)V group had levels characteristic of the control group. In addition, while there was a lack of statistical significance, the CD4+ T cell count for HIV(+)V individuals was 100 cells/*μ*L higher than for those receiving a placebo. In conclusion, the authors of the study reaffirm that the combination of infection with HIV and lifestyle factors typical of injection drug users (for example, a diet which is not rich in antioxidants) may lead to oxidative stress, a potential factor in AIDS development.

In the sections which follow, a mathematical model is developed to investigate the use of antioxidants as a treatment strategy for HIV. We use clinical data from Jaruga *et al. *[28] to estimate parameter values for both control and HIV(+) cases, and then test in detail the results of varying the level of antioxidant supplementation in the HIV(+)V group, largely through numerical bifurcation analysis. We also include an analysis of the sensitivity of our predictions to both parameter estimates and interpatient variability.

### Note

Despite the benefits that can be obtained from antioxidant supplementation, we maintain that the need for accessible and affordable antiretrovirals in developing countries is of utmost importance and must not be neglected.

## Methods

As outlined in the Background, HIV-infected CD4^+ ^T cells can produce HIV virions via two ROS-independent pathways: either directly or through the activation of NF-*κ*B. However, it has been shown that the combined effect of these pathways accounts for a mere one-twentieth of the total virion production [17]. The more substantial fraction of virion production has been attributed to ROS-activated NF-*κ*B [17]. During HIV infection, immune cells (such as macrophages and neutrophils) are also activated, resulting in an increase in ROS generation. Thus, infected cells indirectly produce high levels of ROS, which in turn directly increase the production of virions by infected cells. Antioxidants can control this vicious cycle by reducing ROS concentrations.

**Figure 1:**
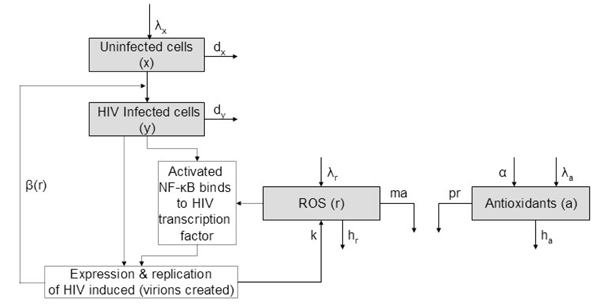
**Schematic diagram of the model**. Reactive oxygen species, while not the sole means for transcription of HIV, directly increase the transcription rate. This results in an increased infection rate *β*(*r*).

To model these processes, we propose a system of differential equations which consists of four populations: uninfected CD4^+ ^T cells (*x*), infected CD4^+ ^T cells (*y*), reactive oxygen species (*r*) and antioxidants (*a*):

where *β*(*r*) is a positive, increasing function. See Figure 1.

### Uninfected CD4^+^

CD4^+ ^T cells are produced by the thymus at constant rate *λ*_*x*_, are eliminated from the system at per-capita rate *d*_*x *_and become infected through mass-action kinetics at rate *β*(1 - ϵ)*xy*, where the infection rate *β *is a function of *r*, described below, and ϵ is the effectiveness of drug therapy.

### Infected CD4^+^

CD4^+ ^T cells become infected at rate *β*(1 - ϵ)*xy*. Infected cells are removed from the system at per-capita rate *d*_*y*_.

### Reactive oxygen species

ROS are naturally produced at constant rate *λ*_*r*_. In the event of infection, ROS are also produced by infected cells at a rate proportional to the number of infected CD4^+ ^T cells, *ky*. ROS are eliminated from the system by reacting with antioxidants at rate *mar *and through all other processes, including reactions with NF-*κ*B and other molecules, such as enzymes, at decay rate *h*_*r*_*r*.

### Antioxidants

Antioxidants are introduced into the system via dietary intake at constant rate *λ*_*a*_. Plasma antioxidant levels may be supplemented therapeutically at constant rate *α*. Antioxidants have natural decay rate *h*_*a*_*a*. Since a large fraction of antioxidants are regenerated after reaction with ROS, we define a new rate of antioxidant consumption, *par*, where *p *is much smaller than *m*.

### Infectivity

To capture ROS-activated transcription in our model, we would like *β*(*r*) to be a saturating, increasing function of *r*. For simplicity, we choose a Michaelis-Menten equation. Therefore, we take

**Figure 2:**
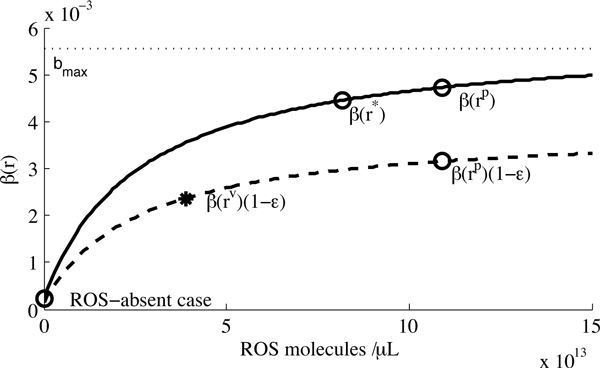
**The *β*(*r*) curve**. The *β*(*r*) curve, denoted by the solid line, represents the rate of infection in the absence of drug therapy. The dashed line depicts the decrease in the infection rate due to HAART, the *β*(*r*)(1 - ϵ) curve. The dotted line represents the maximum rate of infection.

While several other forms of *β*(*r*) might be equally reasonable, this expression provides a good fit to the (limited) data derived from clinical studies (the "ROS-absent", *β*(r*) and *β*(*r*^*p*^) points described in the Parameter Estimation section, and illustrated in Figure 2).

### Note

Many standard HIV models also incorporate an explicit virion population. While virions are not directly modelled in our system, the vital role that they play is not neglected: since they are in quasi-equilibrium with the infected cells, the concentration of virions in the system is roughly proportional to that of the infected cells [36,37].

## Results

### Analytical results

Evaluating for the equilibria yields one biologically meaningful disease-free equilibrium:

where *B *= *λ*_*a*_*m *+ *α*_*m *_+ *h*_*r*_*h*_*a*_-*λ*_*r*_*p*. Thus, when *λ*_*r *_> *h*_*r*_*r*^*d*^, or whenever the production rate of ROS exceeds their overall removal rate, an HIV-negative individual will exhibit a balanced ROS-antioxidant equilibrium.

Using the next-generation matrix method from [38], we find the basic reproductive ratio to be

which makes intuitive sense since a single infected cell at the uninfected equilibrium will produce new infected cells at rate *β*(*r*^*d*^)(1 - ϵ)*x*^*d*^, for mean lifetime 1/*d*_*y*_. (We note that, in practice, ϵ is almost always zero in this situation.)

We next examine stability of the disease-free equilibrium using the following Jacobian:

This yields four eigenvalues

Therefore, the disease-free equilibrium is stable when *R*_0 _< 1 (from (8)).

In addition to the disease-free equilibrium, two biologically meaningful internal equilibria exist; we omit their analytical expressions here since their complicated form offers little insight. Instead, following parameter estimation, we complete a bifurcation analysis of all three biologically meaningful equilibria in the Numerical Results. We note that our model, and the analytical results described up to this point, could be generalized to other factors that are produced in proportion to infected T cells (*ky *term), increase the in-host transmission rate (*β*(*r*) term) and can be counteracted through mass-action kinetics by some exogenous factor (*mar *term). However, in the next section, we estimate parameters specific to ROS and antioxidants, and further numerical results are thus specific to this case.

### Parameter estimation

Developing reasonable (if uncertain) parameter estimates is one of the most difficult aspects of theoretical immunology, and yet can be an extremely worthwhile endeavour [39]. In the tables and subsections which follow, we describe our estimates for both control (HIV(-)) and HIV-positive parameters. We examine the sensitivity of our main results to these estimates in the Sensitivity Analysis.

The model described above includes a total of four populations and 15 parameters. Estimates of six of these parameters (*λ*_*x*_, *d*_*x*_, *d*_*y*_, *λ*_*a*_, *h*_*a *_and ϵ) were directly obtained from the literature and can be found in Table 1. We use *R*_0 _and the seven clinically measured equilibrium levels from Table 2 to deduce the other parameters (see Table 3), except for *α*, which we will vary to investigate therapy. Throughout this section and the numerical work which follows, we will use units of cells per *μ*L plasma (for *x *and *y*) or molecules per *μ*L plasma (for *a *and *r*). In estimating parameters related to the population *r*, we specifically examine the reactive oxygen species hydrogen peroxide as it has been shown to play an important role in the activation of HIV transcription. Moreover, since ascorbic acid (vitamin C) has been cited as a key H_2_O_2 _scavenging antioxidant [40], we use it as our antioxidant for the purpose of parameter estimation.

**Table 1:** Parameter estimates from the literature

Parameter	Value	Reference
*λ*_*x*_	60.76 cells *μ*L^-1 ^day^-1^	estimated from = *λ*_*x*_/*d*_*x *_[49]
*d*_*x*_	0.057 day^-1^	[50]
*d*_*y*_	1 day^-1^	[51]
	5.99 × 10^7 ^day^-1^	(half life = 1 ms) [52]
	5.47 × 10^13 ^molecules *μ*L^-1 ^day^-1^	[5]
*λ*_*a*_	2.74 × 10^13 ^molecules *μ*L^-1 ^day^-1^	estimated from [5]
*h*_*a*_	0.0347 day^-1^	(half life = 8 - 40 days, choose 20 days) [45]
*R*_0_	4.5	estimated from [36]
ϵ		estimated from [43]

**Table 2:** Equilibrium populations from the literature

Parameter	Value	Reference
**Healthy control group**		

	1,066 cells/*μ*L	[49]
	51.5 ± 4.95 *μ*M	[53]
	56.8 ± 4.5 *μ*M	[28]

**HIV(-) (IDU)**		

x*	1,066 cells/*μ*L	[49]
a*	12.1 ± 1.8 *μ*M	[28]

**HIV(+)P (IDU)**		

*x*^*p *^+ *y*^*p*^	360 cells/*μ*L	[28]
*y*^*p*^	43 cells/*μ*L	from equation (10)
*a*^*p*^	8.2 ± 1.8 *μ*M	[28]

**HIV(+)V (IDU)**		

*x*^*v *^+ *y*^*v*^	460 cells/*μ*L	[28]
*y*^*v*^	37 cells/*μ*L	from equation (10)
*a*^*v*^	49.0 ± 5.0 *μ*M	[28]

**Table 3:** Parameter estimates

Parameter	Value
*b*_0_	0.000211 (cell/*μ*L)^-1 ^day^-1^
*b*_max_	0.00621 (cell/*μ*L)^-1 ^day^-1^
*r*_half_	3.57 × 10^13 ^molecules *μ*L^-1^
*h*_*r*_	1.66 × 10^7 ^day^-1^
*λ*_*r*_	1.86 × 10^21 ^molecules *μ*L^-1 ^day^-1^
*K*	1.49 × 10^19 ^molecules cell^-1 ^day^-1^
*M*	1.27 × 10^-6 ^(molecule/*μ*L)^-1 ^day^-1^
*α*	variable molecules *μ*L^-1 ^day^-1^
*P*	5.04 × 10^-14 ^(molecule/*μ*L)^-1 ^day^-1^

In this section and the work which follows, we also refer to four cases of the infected equilibrium, which differ only in their parameter values. Specifically, we denote (1) the uninfected, control diet case with a "hat" (i.e. ), (2) the uninfected, IDU case with an asterisk (i.e. x*), (3) the infected, placebo case with a superscript *p*(i.e. *x*^*p*^) and (4) the infected, vitamin supplementation case with a superscript *v*(i.e. *x*^*v*^) (see Table 2). These populations correspond to the healthy control, HIV(-), HIV(+)P and HIV(+)V groups of Jaruga *et al. *[28], respectively.

#### Literature estimates for  and *λ*_*a*_

It has been recommended that dietary vitamin C intake for all individuals exceed 200 mg per day [41]. Seventy-eight percent, or about 160 mg per day, of this amount is absorbed by the approximately 10 L volume of plasma and extracellular space [42]. This corresponds to an antioxidant introduction rate in the control group, , of 5.47 × 10^13 ^molecules *μ*L^-1 ^day^-1 ^. In order to account for the fact that injection drug users (IDUs) may have a smaller vitamin C intake, we set the amount of dietary vitamin C absorbed in groups HIV(-), HIV(+)P and HIV(+)V to be 80 mg/day which yields *λ*_*a *_= 2.74 × 10^13 ^molecules *μ*L^-1 ^day^-1^. Both of these estimates have a high degree of uncertainty since the pharmacokinetics and bioavailability of ascorbic acid are complex [42]. These parameter values will be examined in the Sensitivity Analysis to follow.

#### Finding *x*^*p*^, *y*^*p*^, *x*^*v *^and *y*^*v*^

The clinical data in Table 2 give only the sum of CD4+ T cells, *x*^*p *^+ *y*^*p *^and *x*^*v *^+ *y*^*v*^. To find each term independently, we combine equation (1) and equation (2), at equilibrium,

where *λ*_*x*_, *d*_*x *_and *d*_*y *_are known. Thus, for the HIV(+)P case, we find *x*^*p *^= 317 and *y*^*p *^= 43. Likewise, for the HIV(+)V case, we find *x*^*v *^= 423 and *y*^*v *^= 37.

#### Estimating the function *β*(*r*) and ϵ

The Jaruga *et al. *study [28] which we use to estimate certain parameters was comprised of HIV-negative individuals and patients on highly active antiretroviral therapy (HAART). Since HAART reduces the rate of infection in an HIV-positive individual, we consider the effectiveness of this therapy in our model, denoted by ϵ. To estimate this parameter, we use the results of a study by Manfredi *et al. *[43] which examined a group of individuals of a similar mean age to those of the Jaruga *et al. *study [28] (33.9 ± 1.6 vs 27 ± 9), the majority of whom were also IDUs [43]. Twelve months of treatment were shown, on average, to increase these patients' CD4^+ ^T cell counts from 231 ± 87 cells/*μ*L to 345 ± 62 cells/*μ*L, which is approximately the same level as in the HIV(+) groups in Jaruga *et al. *[28]. Using the concentration of CD4^+ ^T cells before and during therapy as a proxy to estimate effectiveness, and assuming that this effectiveness has reached equilibrium after twelve months, we set ϵ to be . We note that this overall measure of the effectiveness of therapy includes pharmacological effectiveness, as well as the adherence of the IDU group.

We are ultimately interested in modelling the three IDU populations, HIV(-), HIV(+)P and HIV(+)V. Therefore, we take *R*_0 _to be defined at the HIV(-) case where ϵ = 0. Using (6), we find *R*_0 _at this equilibrium to be:

Given the parameter values in Table 1, this yields *β*(r*) = 0.00422. Since NF-*κ*B activation results in a 20-fold increase in HIV transcription [17], we let *β*(r*) = 20*b*_0 _and thus *b*_0 _= 0.000211. From the disease-free IDU equilibrium we therefore have two points with which to fit the *β*(*r*) curve, *β*(r*) and *β*(0). A third point is obtained from the HIV(+)P equilibrium. In this case, since *y *≠ 0, *d*_*y *_= 1 and *β*(*r*)(1 - ϵ) =  at equilibrium (equation (2)), *β*(*r*^*p*^) =  = 0.00473.

These three points on the *β*(*r*) curve allow us to fit the two other free parameters, yielding *b*_max _= 0.00621 and *r*_half _= 3.57×10^13^. This fixes the function *β*(*r*) (see Figure 2) which models the rate of infection in the absence of drugs. A second curve modelling the effect of therapy, *β*(*r*)(1 - ϵ), can be used to iteratively estimate a further free parameter *h*_*r*_. The procedure we use is to estimate a value of *h*_*r*_, then follow through the steps described for estimating *λ*_*r*_, *p*, *m *and *k*. This allows for numerical estimates of four more parameters and ultimately yields an estimate for *r*^*v*^, the concentration of ROS at the HIV(+)V equilibrium. We then iteratively adjust our initial estimate of *h*_*r *_such that *βr*^*v*^(1 - ϵ) = 1/*x*^*v *^lies along the dashed curve in Figure 2. This procedure yields *h*_*r *_= 1.66 × 10^7 ^day^-1.^

#### Estimating *λ*_*r*_, *p*, *m *and *k*

Given that *m* + *h*_*r *_= 5.99 × 10^7 ^day^-1 ^(Table 1), knowing  and assuming *h*_*r*_, we directly compute *m *= 1.27 × 10^-6. ^In addition, since *y *= 0 at the uninfected equilibrium, equation (3) at equilibrium yields

We assume that *λ*_*r*_, the rate at which ROS are naturally produced, is constant for all individuals. In contrast, *λ*_*a *_represents the dietary influx of antioxidants, and we thus assume that *λ*_*a *_is constant for the HIV(-) and HIV(+) groups, but may differ for the control group. Therefore, we use a* and r* to find *λ*_*a *_for the IDU groups. Thus, we must first find r*. Using (3) at equilibrium,

The parameter *p*, which should be constant for all individuals, is found using (4) at the control equilibrium, i.e., for *y *= 0 and *α *= 0:

From equation (4) at the HIV(+)P equilibrium,

We find our final parameter, *k*, from equation (3) at the HIV(+)P equilibrium:

Finally, from equation (3) at the HIV(+)V equilibrium:

## Numerical results

**Figure 3:**
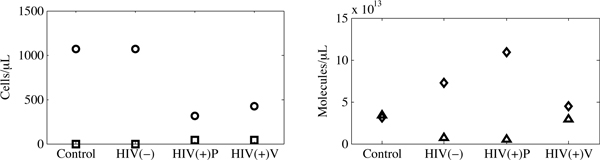
**The analytical results for the control, HIV(-) and HIV(+)P groups**. We include the unstable equilibrium point of the HIV(+)V group at a total vitamin C supplementation level of approximately 116 mg/day. The respective levels of uninfected cells are denoted with circles, infected cells with squares, ROS with diamonds and antioxidants with triangles.

Using the parameters in Tables 1 and 2, the equilibria of our model were found analytically. At these parameter values and antioxidant supplementation levels, only one biologically meaningful internal equilibrium exists, and this equilibrium agrees well with the CD4^+ ^T cell and antioxidant concentrations in Jaruga *et al. *[28], as illustrated in Figure 3. In the first two columns, we compare the control individuals with the HIV-negative IDUs whose lifestyle, including a poorer diet, is a closer control to the HIV-positive IDUs in the Jaruga *et al. *study [28]. As expected, a significant increase in ROS and decrease in antioxidant concentrations is observed in the HIV(-) group. Furthermore, in the presence of HIV infection and absence of antioxidant treatment, these trends continue: the concentrations of ROS and antioxidants further increase and decrease, respectively, in the HIV(+)P group. This is combined with a sizable drop in the total CD4^+ ^T cell concentration from 1066 cells/*μ*L to 360 cells/*μ*L. With daily antioxidant supplementation of approximately 116 mg, the antioxidant concentrations increase and ROS concentrations decrease, but neither quite reach the levels observed in control individuals. Although at this level of supplementation the analytically predicted equilibrium does reach the CD4^+ ^T cell equilibrium of 460 cells/*μ*L found for the HIV(+)V group in Jaruga *et al. *[28], it is important to note that this equilibrium point is unstable, as described in greater detail below.

**Figure 4:**
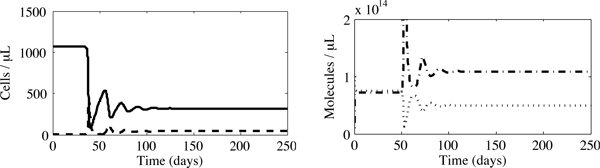
**An initially uninfected IDU who subsequently becomes infected**. Here we observe a significant drop in uninfected CD4^+ ^T cell levels (solid line) characteristic of the infection. An equilibrium is eventually reached where *x *= 317 cells/*μ*L, *y *= 43 cells/*μ*L, *r *= 1.09 × 10^14 ^molecules/*μ*L and *a *= 4.94 × 10^12 ^molecules/*μ*L. The concentration of infected cells is represented by the dashed line and ROS by the dashed-dotted line. For clarity the time course of antioxidants has been scaled; the dotted line plots 10*a*.

Before examining the benefits and limitations of vitamin supplementation, we test our analytical results using numerical integration (MATLAB ^®^, The MathWorks Inc.) for HIV-negative IDUs who subsequently become infected with HIV. In the absence of vitamin supplementation, such an individual would display trends similar to those observed in Figure 4: an initially healthy concentration of CD4^+ ^T cells is followed, upon infection, by a sharp decline in the number of uninfected CD4+ T cells which eventually equilibrates at a significantly lower concentration of 317 cells/*μ*L. In addition, the ROS concentration increases to an equilibrium value well beyond normal levels and the antioxidant concentration decreases. Note that in Figure 4b the antioxidant concentration is scaled by a factor of ten so that these trends can be more clearly observed.

**Figure 5:**
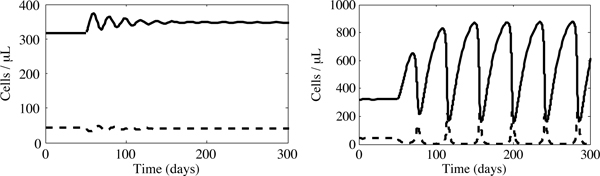
**Uninfected (solid line) and infected (dashed line) cell concentration for an initially infected IDU who begins vitamin supplementation on day 50**. In (a), a stable equilibrium results from a supplement of *α *= 2.0 × 10^13 ^molecules/(*μ*L day) which corresponds to 58 mg of daily vitamin C supplementation. In (b), a periodic cycle appears when *α *= 4.0 × 10^13 ^molecules/(*μ*L day), corresponding to 116 mg of daily vitamin C supplementation.

Next, we examine the behaviour of our model when patients are given moderate daily vitamin supplementation. For this case, our model suggests that an HIV-positive IDU's T cell count can increase, with a concomitant reduction of ROS. However, the magnitude and nature of these changes are dependent upon the level of supplementation. Notice, for example, the outcomes of two different supplementation levels in Figure 5. When we supplement the diet with 58 mg of absorbed antioxidants per day, an increase in the level of uninfected CD4^+ ^T cells (to 345 cells/*μ*L) is observed. However, as we noted in the discussion of Figure 3, we are unable to reach the clinical mean, *x*^*v*^, found in Jaruga *et al. *[28]. Instead, the level of supplementation required for a mean CD4^+ ^count of 460 cells/*μ*L, 116 mg/day, results in the oscillatory dynamics illustrated in Figure 5b.

**Figure 6:**
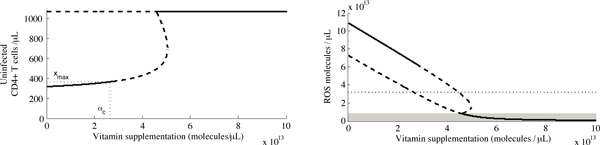
**Bifurcation diagrams of our model of the uninfected T cells and ROS**. A solid line implies a stable equilibrium and a dashed line implies an unstable equilibrium. In (a), we indicate the maximum attainable stable concentration of uninfected cells, *x*_max _(dotted black line). The dotted horizontal line in (b) indicates the ROS level observed in non-IDU control individuals. We denote the ROS concentration for which the disease-free equilibrium becomes stable with the grey box.

We further investigate this interesting behaviour through numerical bifurcation analysis, substituting our parameter values into the analytically-determined eigenvalues of the Jacobian. Using the vitamin supplementation level, *α*, as a bifurcation parameter, we observe that increasing *α *causes an increase in the concentration of uninfected cells and a decrease in ROS concentrations, as expected (Figure 6). However, there exists a critical vitamin supplementation level, *α*_*c *_= 2.63×10^13 ^molecules/*μ*L per day (approximately 78 mg/day), at which the internal equilibrium undergoes a supercritical Hopf bifurcation: the stable internal equilibrium for *α *<*α*_*c *_becomes a stable limit cycle for *α *> *α*_*c *_(Figure 6). Further analysis reveals three additional bifurcations at values of *α *> *α *_*c*_; however, these are of little clinical relevance.

These bifurcation diagrams also confirm what we found in the Analytical Results: the disease-free equilibrium is stable when *R*_0 _< 1. This occurs when *r <*8.16 × 10^12 ^molecules/(*μ*L day) (shaded region in Figure 6b), with *α *≥ 4.59 × 10^13 ^molecules/*μ*L per day, or a total supplementation level greater than 134 mg/day. Our model therefore suggests that there exists a supplementation level at which an HIV(+) individual could theoretically clear all infected cells in plasma. Yet, this only occurs when the concentration of ROS is well below normal levels, and would therefore not be physiologically possible.

**Figure 7:**
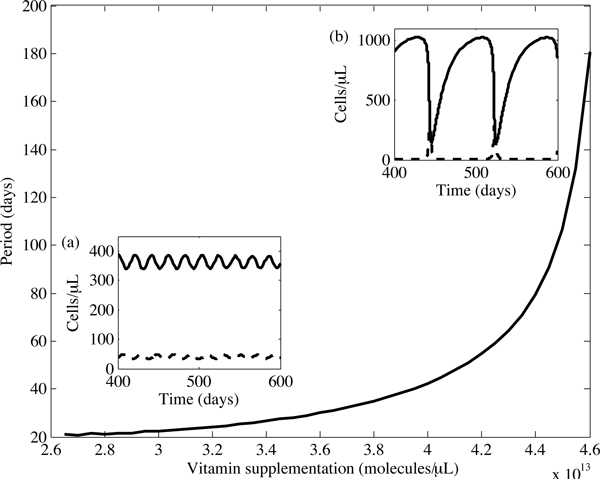
**The period of the limit cycle as a function of vitamin supplementation levels**. As the vitamin supplementation level increases beyond *α*_*c *_= 2.63 × 10^13 ^molecules/*μ*L per day, changes in the dynamics of the limit cycles can be observed. Insets (a) and (b) show the limit cycle at *α *= 2.8 × 10^13 ^and *α *= 4.45 × 10^13^, respectively. In the insets, the uninfected and infected cell concentrations are respectively represented by the solid and dashed lines.

The behaviour of the limit cycle is further examined in the region where *α *> *α*_*c *_by integrating our system numerically for 600 days and measuring the time between the last two peaks. As shown in Figure 7, when vitamin supplementation levels increase above *α*_*c*_, the period of the oscillations increases dramatically. Interestingly, as *α *changes, so does the behaviour of the limit cycle, depicted in the insets of Figure 7. For supplementation levels close to *α*_*c*_, the oscillation is moderate, with symmetrical peaks and troughs. Higher levels of *α*, on the other hand, result in severe oscillations, characterised by extended intervals of high CD4^+ ^T cell counts followed by sharp, short-lived periods in which the patient is in an immunocompromised state. Regardless of the shape of these oscillations, a therapeutic regimen which causes repeated periods of immunosuppression would not be clinically advisable. Thus, our model predicts the existence of a maximum vitamin supplementation level, *α*_*c*_, beyond which further supplementation might be detrimental.

To better understand this threshold behaviour, we look at *x*_max_, which we define to be the maximum attainable stable equilibrium concentration of uninfected T cells; that is, the equilibrium value of *x *when *α *= *α*_*c *_(Figure 6). Using the parameter values as indicated in the Parameter Estimation section, *x*_max _= 369 cells/*μ*L, which falls short of the mean value *x*^*v *^= 423 cells/*μ*L reported in Jaruga *et al. *[28]. To investigate this difference further, we examined the extent to which *x*_max _is sensitive to assumptions regarding our parameter values.

## Sensitivity analysis

We examine the sensitivity of our model to several parameters for which our assumed values have a high degree of uncertainty, or which may display significant interpatient variability. In particular, we look at how the maximum attainable uninfected CD4^+ ^T cell concentration, *x*_max_, changes as a result of varying parameters. In each case, to compute *x*_max_, we performed a numerical bifurcation analysis as illustrated in Figure 6, increasing *α *until the stability of the internal equilibrium is lost.

We test for sensitivity in two ways. First, we examine the sensitivity of *x*_max _to the parameter values from the literature which we initially assumed in the Parameter Estimation section and upon which further parameter estimates depend. In a second analysis, we look at the sensitivity of *x*_max _to interpatient parameter variation. In both sections, we examine the trends in *x*_max _as well as the corresponding concentrations of infected T cells, ROS and antioxidants when a parameter of interest is varied.

### Sensitivity to initial parameter estimates

In this section, we vary five parameters which have a high degree of uncertainty in order to test the overall sensitivity of our results to these assumed parameter values. In cases where the values of other parameters depend on these initial estimates, we subsequently recompute all other dependent model parameters, using the method described in the Parameter Estimation section.

#### Dietary antioxidant intake of the controls

**Figure 8:**
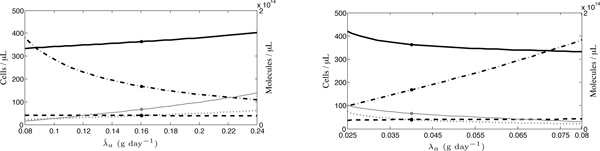
**Sensitivity analyses of (a)  and (b) *λ*_*a*_**. The equilibrium concentration of uninfected cells is represented by the solid line, infected cells by the dashed line, ROS by the dashed-dotted line and antioxidants by the dotted line. We also include the level of antioxidant supplementation, *α*_*c *_(thin, grey line). Levels obtained at the default parameters values (*x*^*v*^, *y*^*v*^, *r*^*v *^and *a*^*v*^) are indicated by dots.

First, due to the natural variability surrounding the diet of control individuals and the uncertainty regarding the amount of antioxidants absorbed, we vary , the amount of antioxidants absorbed from the diet of control individuals. Note that, when  changes, so do our estimates of parameters *h*_*r*_, *m*, *k*, *b*_max_, *r*_half _and *p*. In addition, equilibria r*, *r*^*p *^and *r*^*v *^were altered. From Figure 8a, it may be observed that, as  increases, our model predicts a reduction in *r*^*v*^, while *x*_max _increases only slightly: a 200% increase in  causes a 21% increase in *x*_max_.

#### Dietary antioxidant intake of IDUs

For reasons similar to those posed above, we secondly analyse the sensitivity of *λ*_*a*_, the amount of antioxidants absorbed from the diet of IDUs, and find that *x*_max _decreases modestly as *λ*_*a *_increases (Figure 8b). Note that, when *λ*_*a *_changes, so do our estimates of parameters *h*_*r*_, *m*, *k*, *b*_max _and *r*_half _. Equilibria r*, *r*^*p*^and *r*^*v *^were altered as well. This restricts the range we can examine; when *λ*_*a *_< 0.048 g day^-1^, the positivity of certain parameter values is lost. Importantly, close to the lowest possible value of *λ*_*a*_, we are able to replicate the HIV(+)V Jaruga *et al. *[28] results; that is, *x*^*v *^+ *y*^*v *^= 460.

Again, a very modest change is observed: a 220% parameter increase results in a 21% decrease in *x*_max_.

#### Drug effectiveness

**Figure 9:**
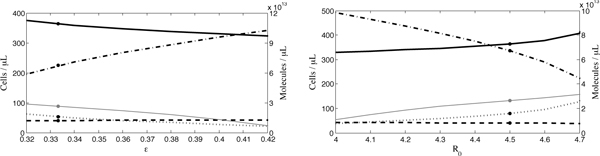
**Sensitivity analyses of (a) ϵ and (b) *R*_0_**. The concentration of uninfected cells is represented by the solid line, infected cells by the dashed line, ROS by the dashed-dotted line and antioxidants by the dotted line. We also include the level of antioxidant supplementation, *α*_*c *_(thin, grey line). Levels obtained at the default parameter values are indicated by dots.

Third, we vary drug effectiveness due to our uncertainty surrounding its estimate and its dependence upon the treatment regimen. When ϵ changes, so do our estimates of parameters *h*_*r*_, *m*, *k*, *R*_0_, *b*_max _and *r*_half _. Equilibria r* and *r*^*v *^were altered as well. In Figure 9a, an increasing ϵ is shown to yield a decreasing *x*_max_, although again *x*_max _is moderately sensitive to this parameter: a 31% increase in ϵ causes a 14% decrease in *x*_max_. Note that, at higher values of ϵ than illustrated in Figure 9a, the stability of the internal equilibrium is lost, whereas at lower values the positivity of certain parameters is lost. This restricted range of ϵ only applies to our estimates of mean drug effectiveness for the IDU group in the Jaruga *et al. *[28] study; interpatient variation in ϵ is possible over a much wider range, as described in detail below.

#### Basic reproductive ratio

Fourth, since there is uncertainty surrounding the value of *R*_0_, the results of a range of parameter values are analysed. Note that, when *R*_0 _changes, so do our estimates of parameters *h*_*r*_, *m*, *k*, *R*_0_, *b*_max _and *r*_half _. Equilibria r* and *r*^*v *^were altered as well. We observe in Figure 9b that, as *R*_0 _increases by 17%, *x*_max _increases by 24%; therefore, we find that *x*_max _is somewhat sensitive to changes in *R*_0_. Values of *R*_0 _lying below the range presented in Figure 9b cause the disease-free equilibrium to regain stability, whereas those that are higher result in negative parameter values.

#### ROS removal

Finally, since the removal rate of ROS is extremely rapid and is therefore difficult to compute, we analyse the system for varying removal rates, . We find that our results are completely insensitive to changes in  (data not shown), since the values of the subsequently computed parameters, namely *h*_*r*_, *m *and *k*, exactly compensate for this change.

Despite these cascading changes to subsequently computed parameters in response to changes in , *λ*_*a *_or ϵ, we find that *x*_max _is fairly insensitive. However, the value of *x*_max _is somewhat sensitive to our initial assumption of the in-host *R*_0 _for HIV, which is interesting given that the value of this parameter is not well known [36]. In contrast, the predicted ROS concentration at *α *= *α*_*c *_is very sensitive to our initial assumptions regarding these parameters. We are able to replicate clinical results under the assumption that the IDU group has a very low dietary intake of antioxidants, corresponding to 48 mg absorbed per day.

### Sensitivity to interpatient variability

In this section, we quantify the sensitivity of our model to interpatient variation for several parameter values. Unlike in the previous section where dependent parameter values were recalculated in response to variation in an assumed parameter, here we only vary the parameter of interest and hold all other parameters constant, except *α *which we vary in order to find *α*_*c *_as before.

#### Drug effectiveness

**Figure 10:**
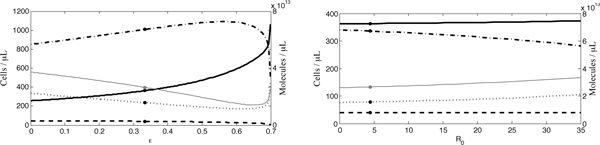
**Sensitivity analyses of (a) ϵ and (b) *R*_0 _for interpatient variability**. The concentration of uninfected cells is represented by the solid line, infected cells by the dashed line, ROS by the dashed-dotted line and antioxidants by the dotted line. We also include the level of antioxidant supplementation, *α*_*c *_(thin, grey line). Levels obtained at the default parameter values are indicated by dots.

Our first parameter of interest is drug effectiveness, since ϵ varies from patient to patient due to differences in HIV progression and levels of adherence. As anticipated, our model is sensitive to the level of effectiveness, with *x*_max _(solid line) rising with increasing effectiveness (Figure 10a). Furthermore, our model suggests that increasing a patient's drug effectiveness from  to 0.7 is sufficient to drive the plasma concentration of infected cells to undetectable levels, as is observed in aggressive HAART [44]. Increased drug effectiveness also results in a reduction in the level of antioxidant supplementation required to realize *x*_max _and increases the chance of oversupplementation. Thus, our model predicts, interestingly, that antioxidant supplementation should be reduced in patients who exhibit strong adherence, although some level of supplementation would continue to be beneficial.

#### Basic reproductive ratio

Second, we test the sensitivity of our results to *R*_0_, since this parameter could also display interpatient variability due to differences in immunocompetence, disease progression and other factors. As we observe in Figure 10b, although the concentration of ROS decreases, *x*_max _is relatively insensitive to changes in *R*_0 _over an extremely wide range: an increase from 0 to 35 results in a mere 3% increase in *x*_max_.

#### Natural ROS production

**Figure 11:**
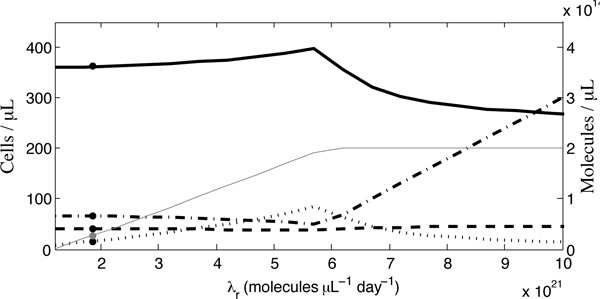
**Sensitivity analysis of *λ*_*r *_for interpatient variability**. The concentration of uninfected cells is represented by the solid line, infected cells by the dashed line, ROS by the dashed-dotted line and antioxidants by the dotted line. We also include the level of antioxidant supplementation, *α*_*c *_(thin, grey line). Levels obtained at the default parameter value are indicated by dots. Stability is lost for lower values of *λ*_*r *_than those illustrated.

In the formulation of our model, we made the assumption that the natural rate of ROS production, *λ*_*r*_, was the same for all individuals. Therefore, we thirdly examine the effect of a varying interpatient *λ*_*r*_. In Figure 11, it may be observed that, despite an increasing *λ*_*r*_, ROS concentrations (dashed-dotted line) initially decrease and therefore *x*_max _(solid line) initially increases. This trend can be attributed to significant increases in antioxidant supplementation levels (thin, grey line); as *λ*_*r*_increases, higher values of *α*_*c *_are possible without losing the stability of the equilibrium. However, since physiological constraints would presumably impose some limit on the degree of the vitamin supplementation possible, we set a maximum antioxidant supplementation level of 2.0 × 10^14 ^molecules *μ*L^-1 ^day^-1^, which is approximately 586 mg/day, absorbed into the bloodstream. The quantitative value of this limit has been chosen arbitrarily to illustrate the qualitative effects of the physiological limit which presumably exists.

Thus, the increases in *x*_max _continue until *α *reaches our imposed maximum, which in this example occurs when *λ*_*r *_= 5.65 × 10^21 ^molecules *μ*L^-1 ^day^-1^. Further increasing *λ*_*r*_, combined with a constant *α *level, results in a significantly increasing ROS concentration which causes *x*_max _to decrease. We address this interesting qualitative prediction further in the Discussion.

#### Dietary antioxidant intake of IDUs

Lastly, we examine the effect of a varying dietary antioxidant intake and find that our results are insensitive to this variation, the only change being an alteration in the vitamin supplementation level required to achieve *x*_max _(data not shown).

## Discussion

We have developed and analysed a simple model of the interactions between CD4^+ ^T cells, reactive oxygen species and antioxidants. Verifying the results of various clinical studies, our model predicts that moderate levels of antioxidant supplementation in HIV-positive IDUs can lead to an increase in uninfected CD4^+ ^T cell concentrations. However, our model also suggests that excessive supplementation could cause fluctuating T cell concentrations in these individuals. For example, consider the limit cycle in Figure 5b: in this case, a patient's immunological response is periodically compromised - characterized by a low concentration of uninfected CD4^+ ^T cells - leaving the individual vulnerable to opportunistic infections.

**Figure 12:**
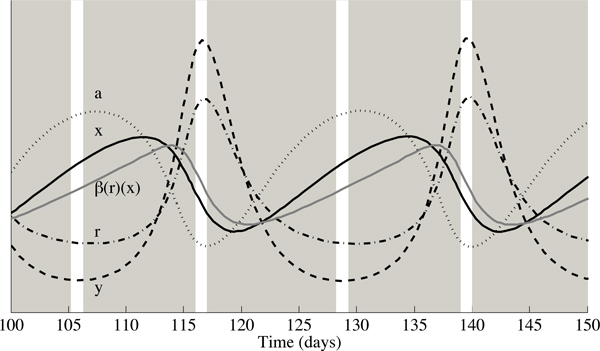
**A closer look at the dynamics of the stable limit cycle**. The concentration of uninfected cells is represented by the solid black line, infected cells by the dashed line, ROS by the dashed-dotted line and antioxidants by the dotted line, each rescaled for comparison. The solid grey line denotes *β*(*r*)*x*. White vertical bars delineate maxima and minima in infected cell concentrations.

In an effort to understand this periodic behaviour, we take a closer look at the system dynamics when the level of antioxidant supplementation is above the critical level, *α*_*c*_, in Figure 12. In this figure, populations during the limit cycle are rescaled to facilitate comparison, while white vertical bars delineate the peak and trough concentrations of infected T cells. The most direct result of an increase in antioxidant supplementation is first an increase in the antioxidant concentration (dotted line) and a decrease in ROS (dashed-dotted line). These two effects produce a concomitant increase in uninfected cells (solid, black line) and reduction in infected cells (dashed line). As the concentration of uninfected cells increases, the infection rate per infected cell (*β*(*r*)*x*, grey line), reaches high levels, allowing both infected cell and ROS concentrations to increase sharply. These increases are short lived in part because of the extremely short half-life of ROS, and due to a rapid reduction in *β*(*r*)*x*. As ROS and infected cell concentrations plummet, the cycle is allowed to repeat. One hypothesis is that when the level of antioxidant supplementation is too high, the infection rate *β*(*r*)*x *reaches too high a peak to allow for a stable equilibrium.

Regardless of its cause, the appearance of a limit cycle in our model could explain why some clinical studies show no improvement in patients' average CD4+ T cell concentrations: it is plausible that high supplementation levels could cause fluctuating T cell counts which are then sensitive to the details of measurement timing, leading to the conclusion that antioxidant supplementation has no immunological benefit for HIV-positive patients.

Since antioxidant supplementation levels above a critical value, *α*_*c*_, have the potential to pose difficulties for patients, we turn our attention to the stable equilibria obtained when *α *<*α*_*c*_. We examined in particular the maximum concentration of uninfected CD4^+ ^T cells, *x*_max_, which could be obtained in principle as a stable equilibrium via antioxidant supplementation. We found *x*_max _to be relatively insensitive to moderate variation in five initial parameter estimates, particularly when subsequent parameter estimates were changed as a result of these alternative assumptions. This insensitivity is presumably because subsequent parameters act to compensate for alternative assumptions, since we set parameters to match the clinically-observed equilibria. These compensatory changes also explain why the results described in the analysis of the sensitivity to initial parameter estimates seem counter-intuitive; for example, as our initial assumption for the in-host *R*_0 _increases, *x*_max _also increases (Figure 9b). In contrast, interpatient variability results in a higher degree of sensitivity for certain parameters, as expected. We note that *x*_max _is unlikely to be achieved in practice, since the required level of precision in the supplementation level would be impossible.

**Figure 13:**
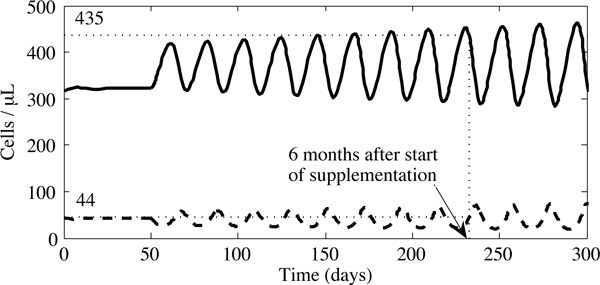
**The oscillatory dynamics of the system when 84 mg of the daily vitamin supplement is absorbed**. We see that six months after the start of supplementation, we reach CD4^+ ^T cell levels observed in the Jaruga *et al. *study. The concentration of uninfected cells is represented by the solid black line, infected cells by the dashed line.

Interestingly, our sensitivity analysis revealed that even when our initial parameter estimates were varied, the mean T cell count observed by Jaruga *et al. *[28] after six months of antioxidant therapy was higher than any stable equilibrium value predicted by our model, except when considering exceptionally low values of *λ*_*a*_. In the region of instability, however, values equivalent to the clinical data were frequently observed. For example, in Figure 13, we present an example in which the sum of uninfected and infected CD4^+ ^T cells at six months, 479 cells/*μ*L, exceeds the 460 cells/*μ*L found in Jaruga *et al. *[28]. This was achieved with our default parameters and antioxidant supplementation of 2.87 × 10^13 ^molecules/*μ*L per day, or about 84 mg of absorbed antioxidants per day. This outcome is anecdotal and highly dependent upon the amount of vitamin C absorbed; however, it illustrates the potential sensitivity of clinical results to the details of measurement timing.

To further investigate the benefits of antioxidant supplementation, we hope that future work could see the model extended to include appropriate pharmacokinetics of antioxidants. In its present form, our model considers *α *to remain constant over time. If we included the full dynamics of antioxidant concentrations after an oral dose, including varying the antioxidant decay rate with plasma concentration [45], we predict that the oscillatory behaviour observed here would be exacerbated. Either standard pharmacokinetic modelling [46] or impulsive differential equations [39] could be used to examine such effects.

It would also be interesting to explore the effects of enzymatic antioxidants: glutathione peroxidase and catalase, for example. Both of these enzymes are used in the elimination of hydrogen peroxide (H_2_O_2_), but are not consumed by these reactions. Their short half-lives (less than 10 minutes) [47,48], however, could further exacerbate the variability already observed in the simple model.

## Conclusion

While antioxidant supplementation may not be a long term solution for HIV-positive IDUs, our model suggests that moderate doses of antioxidants may temporarily boost uninfected CD4^+ ^T cell concentrations. This might enable HIV-positive individuals to lengthen the interval before costly drugs with severe side effects become necessary. These results could have implications for infected individuals in HIV-endemic areas, since dietary antioxidant intake depends on the availability of adequate antioxidant-rich produce. Moreover, where access to antiretroviral therapy is limited or non-existent due to economic constraints, a significantly more affordable vitamin supplementation therapy could potentially provide some limited benefit. Of course we emphasize that this in no way reduces the need for accessible and affordable antiretrovirals in developing countries.

## Competing interests

The authors declare that they have no competing interests.

## Authors' contributions

RDvG and LMW developed the model. RDvG analyzed the model, analytically and numerically, and produced all figures. RDvG and LMW interpreted the results. RDvG drafted the manuscript.
